# Insights from the yield, protein production, and detailed alkaloid composition of white (*Lupinus albus*), narrow-leafed (*Lupinus angustifolius*), and yellow (*Lupinus luteus*) lupin cultivars in the Mediterranean region

**DOI:** 10.3389/fpls.2023.1231777

**Published:** 2023-12-15

**Authors:** Inês M. Valente, Carla Sousa, Mariana Almeida, Carla Miranda, Victor Pinheiro, Sofia Garcia-Santos, Luís M. M. Ferreira, Cristina M. Guedes, Margarida R. G. Maia, Ana R. J. Cabrita, António J. M. Fonseca, Henrique Trindade

**Affiliations:** ^1^ REQUIMTE, LAQV, ICBAS, School of Medicine and Biomedical Sciences, University of Porto, Porto, Portugal; ^2^ REQUIMTE, LAQV, Departament of Chemistry and Biochemistry, Faculty of Sciences, University of Porto, Porto, Portugal; ^3^ Veterinary and Animal Research Centre (CECAV) and Associate Laboratory for Veterinary and Animal Science (AL4AnimalS), Universidade de Trás-os-Montes e Alto Douro, Quinta de Prados, Vila Real, Portugal; ^4^ Centre for the Research and Technology Agro-Environmental and Biological Sciences (CITAB), Universidade de Trás-os-Montes e Alto Douro, Vila Real, Portugal

**Keywords:** crop valorization, Fabaceae, narrow leaf lupin, yellow lupin, white lupin

## Abstract

**Introduction:**

Lupins and other legumes have been considered as alternative plant-based protein sources to soybeans for both humans and livestock. Furthermore, they can contribute to more sustainable agricultural systems. The productivity and chemical composition of legumes is highly variable between species, cultivars, and with the edaphoclimatic conditions.

**Methods:**

This work evaluated the adaptability of seven *Lupinus* cultivars in two different sowing locations, during two consecutive years, through the characterization of their seed, as a means of investigating their suitability to be used as a source of food and/or feed.

**Results and discussion:**

*Lupinus angustifolius* cv. Tango and *Lupinus luteus* cv. Acos were the most stable genotypes across the environments when considering the seed and protein production, while *L. luteus* cv. Alburquerque and *L. luteus* cv. Mister showed less variation in the total alkaloid content across the environments. The edaphoclimatic conditions affected seed and protein yields, as higher rainfall resulted in high productivity. The lower temperatures observed in the first year at both locations caused a reduction in the production of alkaloids in *L. luteus* cv. Acos and Cardiga. Due to the high alkaloid content of some of the studied cultivars their use as food or feed can pose some safety concerns. However, these cultivars can have high levels of resistance to herbivore and insect attacks, which can be of the utmost importance for the use of these crops for recovering poor or exhausted soils.

## 1 Introduction

Grain legumes (Fabaceae) are valuable sources of plant-based protein for both humans and livestock, and can contribute to more sustainable and healthier low-input agricultural systems due to their ability to fix biological nitrogen through symbiotic association with soil bacteria (Ferreira et al., 2021). Despite the efforts of the European Commission to promote the production of legumes to reduce the historic dependence on imported plant protein sources (European Commission, 2018), these plants continue to be underrepresented in European cropping systems (Watson et al., 2017). However, advances achieved in recent decades regarding breeding, cultivation practices, and policy initiatives (Berger and Ludwig, 2014; MacWilliam et al., 2014; Ferreira et al., 2021) have been promoting their production and consumption. Jensen et al. (2021) have recently reviewed the European legume crop production status and concluded that an annual increase of 1% in legume yields would have a greater impact on protein production in the EU in the medium term than additional subsidies (i.e., 75€ ha^−1^ for soybean and leguminous crops). These figures highlight the need for a continued increase in grain legume productivity through research and development (R&D) to efficiently improve the resilience and economic sustainability of European agrifood systems.

The productivity of Fabaceae varies greatly between species and with soil and climatic conditions (French et al., 2001; Palta et al., 2004; Fraser et al., 2005). Legume crops are particularly vulnerable to extreme temperatures and drought stress during vegetative and reproductive growth (Khatun et al., 2021), which can negatively affect plant development and grain production (Savita et al., 2020). Therefore, it is of the utmost importance to identify the most appropriate legume species and cultivars for specific locations to maximize grain productivity and protein production, particularly in the Mediterranean region where the periods of severe and extreme drought and heat events have been exacerbated by climate change.

White (*Lupinus albus* L.), blue, or narrow-leafed (*Lupinus angustifolius* L.), and yellow (*Lupinus luteus* L.) lupins are native European grain legumes, which are well adapted to acidic and sandy soils, a trait that is of particular importance in many Mediterranean regions and that differentiates them from other grain legumes (Lema and Soengas, 2023). Lupin seeds accumulate proteins preferentially to oils or starch, being a well-balanced protein source, except for the low levels of sulfur-containing amino acids (Musco et al., 2017). Thus, lupins are an interesting source of protein for food and feed, an alternative to soybean, which is also deficient in sulfur-containing amino acids (Lucas et al., 2015; Monteiro et al., 2021). However, lupins, principally if not genetically selected, can accumulate considerable amounts of nitrogenous secondary metabolites, particularly quinolizidine and piperidine alkaloids, which are synthesized from the amino acid lysine, and to a minor extent, indole alkaloids, which are synthesized from tryptophan (Wink et al., 1995; Adhikari et al., 2012).

The alkaloids are a large group of secondary metabolites that plants produce to defend themselves against a variety of pathogenic microorganisms and predators, including insects and herbivores, and against competing plants via allelopathy (Wink and Mohamed, 2003). Alkaloid synthesis occurs predominantly in the aerial green parts of the plant, under light stimulation, and are translocated in the phloem to other parts of the plant, accumulating in seeds as they mature (Otterbach et al., 2019; Mancinotti et al., 2022). The biosynthesis of quinolizidine alkaloids begins with the decarboxylation of L-lysine to cadaverine that forms the nitrogen-containing heterocycle and then the various quinolizidine skeletons of bicyclic, tricyclic, and tetracyclic alkaloids (Frick et al., 2017; Mancinotti et al., 2022). The quinolizidine alkaloids can then be further modified by dehydrogenation, oxygenation, hydroxylation, glycosylation, or esterification to form a wide variety of structurally related quinolizidine alkaloids (reviewed in Frick et al., 2017). Cadaverine is also the precursor in the biosynthesis of piperidine alkaloids (Sato et al., 2018).

The interactions between genotype and environment are complex and alkaloids in lupins can reach considerable levels, thus rendering the plant toxic and with a bitter unpleasant taste to herbivores (Frick et al., 2017). The mechanisms of toxicity and susceptibilities to individual lupin alkaloids include anticholinergic effects and inhibition of voltage-dependent ion channels, with risk of paralysis, spasms and tremors, respiratory failure, and heart arrhythmia; the last underlying the well-established antiarrhythmic effect of sparteine (Schrenk et al., 2019). The piperidine alkaloid ammodendrine is suspected to be teratogenic and some quinolizidine alkaloids may become teratogenic in bovine animals after metabolization in the rumen (Green et al., 2012).

In this study, the adaptability and the production of grain and protein yields of seven cultivars of three *Lupinus* species (i.e., *L. albus*, *L. angustifolius*, and *L. luteus*) in two different sowing locations, during two consecutive years, were evaluated. Detailed alkaloid profiles were also determined to evaluate the impact of cultivars and environmental factors, such as soil characteristics, rainfall, and temperature, on alkaloid biosynthesis. The generated knowledge will contribute to the promotion of lupin production by identifying the best cultivar for use as a source of food and/or feed.

## 2 Materials and methods

### 2.1 Trial location, plant material, experimental design, and environment characterization

Seven cultivars of legume seeds of the genus *Lupinus* were acquired from local seed providers: *L. albus* cv. Dulce and *L. luteus* cv. Alburquerque were supplied by Cicytex—Centro de Investigaciones Científicas y Tecnológicas de Extremadura, Spain; *L. albus* cv. Estoril, *L. angustifolius* cv. Tango, and *L. luteus* cv. Mister were supplied by Fertiprado, Portugal; *L. luteus* cv. Acos and *L. luteus* cv. Cardiga were supplied by INIAV—Instituto Nacional de Investigação Agrária e Veterinária, Elvas, Portugal.

This study was carried out simultaneously over two consecutive years at two different experimental fields on the northeast of Portugal. One site was at Mirandela (MI; 41.511896, −7.161595) and the other at Vila Real (VR; 41.284747, −7.738875). In both years and locations, soils were plowed before sowing and samples (0 cm–30 cm depth) were taken for general physicochemical composition determinations (**Table 1**). Due to soil chemical composition, 1,600 kg of dolomitic lime and 800 kg of superphosphate were added to the soil in VR in the first year and 25 kg was added to the soil on the same site in the second year.

**Table 1:** Soil characteristics and climacteric conditions of MI and VR on both years of the study*.

	MI		VR	
	Year 1	Year 2	Year 1	Year 2
Soil characteristics				
*Particle size distribution (g kg^−1^)*				
Sand	715 ± 3		599 ± 1	
Silt	160 ± 1		275 ± 1	
Clay	125 ± 2		127 ± 1	
*Chemical parameters*				
pH (water)	6.10 ± 0.02		4.75 ± 0.01	
pH (KCl)	5.22 ± 0.05		3.85 ± 0.01	
Organic matter (g kg^−1^)	10.9 ± 0.4		14.0 ± 0.12	
Extractable P (mg P_2_O_5_ kg^−1^)	223 ± 5		67.1 ± 1.1	
Extractable K (mg K_2_O kg^−1^)	114 ± 3		85.6 ± 1.4	
*CEC (cmolc kg^−1^)*				
Al	nq		0.677 ± 0.016	
Ca	4.80 ± 0.07		2.51 ± 0.07	
K	2.69 ± 0.80		0.263 ± 0.002	
Mg	1.09 ± 0.01		0.678 ± 0.029	
Total CEC	6.28 ± 0.09		4.25 ± 0.05	
*Climatic variables*				
Mean minimum temperature (T min)	6.75	5.68	6.71	5.63
Mean maximum temperature (T max)	18.2	16.4	17.0	14.9
Mean temperature (T)	12.5	11.0	11.8	10.3
Mean temperature during spring (T spring)	15.0	12.5	14.2	11.4
Total spring rainfall (R spring)	98.4	327	122	485
Total rainfall (R)	323	668	456	920

CEC, cation exchange capacity; Ca, calcium; cmolc kg^−1^, centimole positive charge per kg of soil; Al, aluminum; Na, sodium; K, potassium; Mg, magnesium; nq, not quantified.

Climatic variables correspond to the experimental period; spring refers to the months of March to May.

The legume cultivars were sown on 28 November 2016 at MI and 23 November 2016 at VR (year 1) and on 15 November 2017 at MI and 21 November 2017 at VR (year 2), using a randomized block design of plots of 10 m^2^ (2.5 m × 4 m). The plots were replicated four times, thus resulting in 28 plots on each location, for each year. The plots were rain fed, no supplemental irrigation was provided. The sowing densities were calculated to achieve crop plant densities of 30 plants m^−2^, 58 plants m^−2^, and 58 plants m^−2^, respectively, for *L. albus, L. luteus*, and *L. angustifolius*. The seeds were placed in rows 30 cm apart, and seed-to-seed distance in rows was calculated based on the targeted crop plant densities above. In addition, the seed-to-seed distance was corrected for the germination rate of each cultivar, which was determined by previous germination tests conducted at the laboratory. The germination rates and the sowing densities for both years are presented in **Table S1** (**Supplementary Information**). The harvest occurred when all the pods in the same plot were ripe on the following dates: MI year 1, between 31 May 2017 and 22 June 2017; VR year 1, between 16 June 2017 and 6 July 2017; MI year 2, between 6 July 2018 and 17 July 2018; and VR year 2, between 12 July 2018 and 20 July 2018. The pods were collected and transported to the laboratory where seed threshing was performed by hand. The grain yield (t DM ha^−1^) was evaluated using a central area of 2 m^2^.

To better characterize each location, daily data on the average minimum and maximum temperatures (˚C) and precipitation (mm) were collected from local weather stations to calculate monthly values from September to August on both growing years (**Figure S1**). The temperature and precipitation data for the study of the environmental effect on productivity and alkaloid composition are provided in **Table 1**.

### 2.2 Proximate chemical composition

The grain seeds were dried in a forced-air oven at 65°C for 24 h for dry matter (DM) determination and ground to pass through a 1-mm sieve. The ground samples were analyzed according to the Association of Official Analytical Chemists (AOAC)’s (AOAC, 2000) methods for DM (934.01), ether extraction (920.39), and Kjeldahl N (954.01) content. The crude protein content was calculated as Kjeldahl N multiplied by a conversion factor (6.25). Neutral detergent fiber content was also determined according to Robertson and Soest (1981) and expressed exclusive of residual ash.

### 2.3 Alkaloid extraction

The alkaloids were extracted from the powdered seeds according to the procedure described by Magalhães et al. (2017). Briefly, alkaloids were extracted with 5% trichloroacetic acid for 30 min, under constant stirring. After centrifugation and alkalinization of the supernatant the crude extract was purified by liquid–liquid extraction with dichloromethane, the organic solvent was evaporated, and the alkaloid-rich residue was stored at −20°C, protected from light, until analysis. Each sample was extracted in duplicate.

### 2.4 GC-MS analysis

The alkaloid extracts were dissolved in dichloromethane and filtered with a 0.45-µm regenerated cellulose syringe filter before gas chromatography–mass spectrometry (GC-MS) analysis, following the conditions described by Magalhães et al. (2017) with modifications. The chromatographic analysis of the extracts was performed in a Thermo Fisher Scientific (Waltham, MA, USA) Trace 1300, ISQ single-quadrupole mass spectrometer equipped with a TraceGOLD TG-5MS column (30 m × 0.25 mm; 0.25 µm) from Thermo Fisher Scientific. The oven temperature was programmed as follows: 150°C for 1 min; followed by an increase at 5°C min^−1^ until the temperature reaches 235°C, it is then held for 15 min; and then the temperature is increased at 10°C min^−1^ until 280°C is reached, then it is held for 10 min. The injection volume was 1 µL and a split ratio of 1:5 was used. The identification of the compounds was performed by the analysis of standards or by comparison with the National Institute of Standards and Technology (NIST)’s database (2001).

### 2.5 Alkaloid quantification

The quantification of each alkaloid in the extracts was achieved by using calibration curves of standards prepared in dichloromethane and analyzed under the same conditions as the samples. The total peak area was plotted as a function of concentration. Gramine (99%; Sigma, St. Louis, MO, USA), lupinine (100%; Sigma), sparteine (≥ 98%; Sigma), angustifoline (> 98%; Ambinter, Orléans, France), and lupanine (> 98%; Ambinter) were quantified as themselves. The other alkaloids were quantified as equivalents of the standard from the same chemical class (indole, piperidine, bicyclic, tricyclic, or tetracyclic quinolizidine).

### 2.6 Statistical analysis

The statistical analyses to evaluate the effect of the environment (represented by the interaction location × year) on the genotype (cultivar) productivity and alkaloid content were performed through the analysis of variance (ANOVA) using the “stability” package (version 0.5.0) in R (software version 4.3.1; The R Foundation for Statistical Computing, Vienna, Austria). The significance was set for *p*-values lower than 0.05 and multiple comparisons of means was carried out using the Tukey test with the packages “multcomp” (version 1.4–25) and “multcompView” (version 0.1–9) in R. The stability of the genotypes across the environments was calculated using the coefficient of variation (CV), the ecovalence stability index (W) (Wricke, 1962; Wricke, 1964), and Shukla’s stability variance (Shukla) (Shukla, 1972). For all the indexes, the lower the value, the higher the stability of the genotype. The correlation pattern between the environmental variables and the environments was characterized via a principal component analysis (PCA) and the two PCA axes were displayed in a distance biplot performed using the packages “FactoMineR” (version 2.8) and “factoextra” (version 1.0.7) in R. The interaction effects between the genotype and the environment as a function of the environmental variables was studied by a redundancy analysis (RDA) using the package “vegan” (version 2.6–4) in R.

## 3 Results

### 3.1 Productivity and proximate chemical composition

The seed and protein yields were affected by the genotype, the environment, and the interaction genotype × environment (**Table 2**; *p* < 0.05), with the environment being the main factor responsible for the variability (73% and 74%, respectively) of the results.

**Table 2:** Analysis of variance of seed and protein production (t DM ha^−1^), and seed chemical composition (g 100 g^−1^ DM) for *Lupinus* cultivars sown in four environments (with a combination of sowing locations and years)*.

Source of variation	df	Production						Composition											
		Seeds			Protein			Ash			Crude protein			Ether extract			Neutral detergent fiber		
		SS	*p*-value	%TRT	SS	*p*-value	%TRT	SS	*p*-value	%TRT	SS	*p*-value	%TRT	SS	*p*-value	%TRT	SS	*p*-value	%TRT
Genotype	6	18.39	< 0.001	11	2.47	< 0.001	10	11.42	< 0.001	57	1,788.22	< 0.001	86	159.41	< 0.001	78	840.66	< 0.001	83
Environment	3	125.82	< 0.001	73	18.86	< 0.001	74	5.35	< 0.001	27	54.38	0.359	3	19.57	0.144	10	18.47	0.243	2
Rep(environment)	12	9.80			1.89			2.04			184.52			35.95			46.37		
Genotype × environment	18	27.94	0.005	16	3.99	0.007	16	3.39	0.031	17	236.04	0.313	11	24.32	0.525	12	157.94	0.274	16
Residuals	68	43.69			6.49			6.76			764.09			96.73			490.80		

*df, degree of freedom; SS, sum of squares; TRT, total sum of squares relative to the main effects (TRT = SS_Environment_ + SS_Genotype_ + SS_Genotype × environment_).

**Figure 1:**
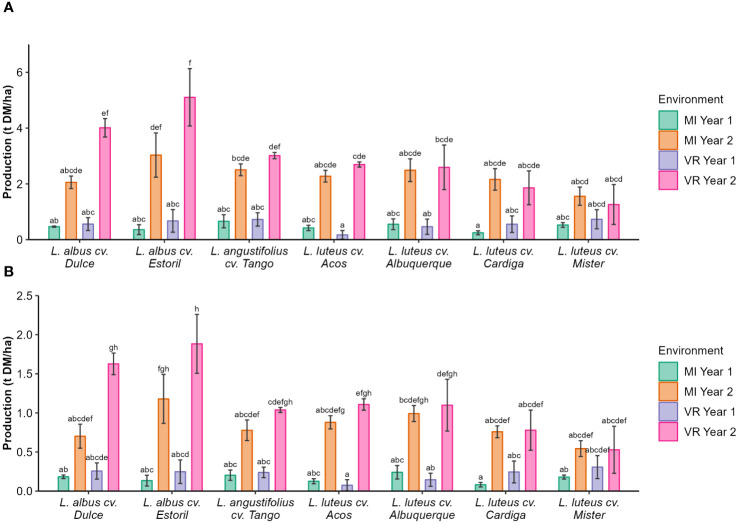
**(A)** Seed and **(B)** protein production (t ha^−1^ DM) by genotype and environment. The different letters show statistically significant differences (*p* < 0.05).

The seed production was lower in the first year of the study, regardless of the cultivar, and seed production was the highest for *L. albus* cv. Estoril in VR year 2 (5.10 t DM ha^−1^; **Figure 1A**). Protein production followed the same pattern as of seed production, that is, the lowest value was found for cultivars in year 1 (0.07 t DM ha^−1^–0.31 t DM ha^−1^; **Figure 1B**) and the highest value was recorded in year 2 for *L. albus* cv. Estoril (1.88 t DM ha^−1^; **Figure 1B**).

Concerning the chemical composition of the *Lupinus* seeds, ash, crude protein, ether extract, and neutral detergent fiber contents varied significantly between cultivars (*p* < 0.001; **Table 2**). Ash content was affected by the interaction genotype × environment (*p* = 0.031), and the ether extract content by the environment (*p* = 0.004; **Table 2**). The ash content varied between 3.60 g 100 g^−1^ DM and 5.28 g 100 g^−1^ DM (**Figure S2**, **Supplementary Information**). The crude protein content was the lowest in *L. angustifolius* cv. Tango (32.2 g 100 g^−1^ DM), followed by *L. albus* cv. Estoril (37.8 g 100 g^−1^ DM), and did not differ (*p* > 0.05) between the other studied cultivars (**Figure S3A**). *Lupinus albus* cultivars presented with the highest ether extract content (7.24 g 100 g^−1^ DM –7.28 g 100 g^−1^ DM; **Figure S3B**). The cultivars of *L. angustifolius* and *L. luteus* presented with the highest neutral detergent fiber content (25.2 g 100 g^−1^ DM–28.4 g 100 g^−1^ DM) and *L. albus* cultivars the lowest (20.5 g 100 g^−1^ DM–20.7 g 100 g^−1^ DM; **Figure S3C**).

### 3.2 Total alkaloid content and by chemical class

**Figure 2:**
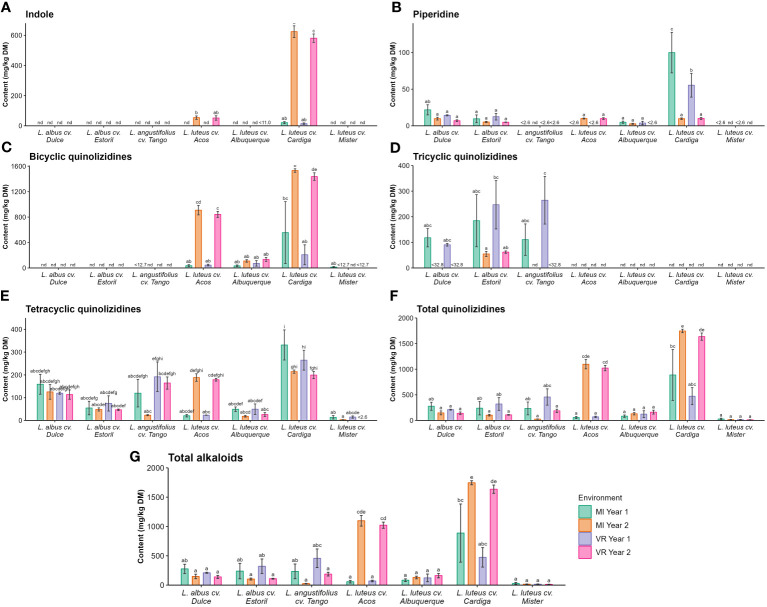
Concentrations (mg kg^−1^ DM) of **(A)** indole, **(B)** piperidine, **(C)** bicyclic, **(D)** tricyclic, and **(E)** tetracyclic, **(F)** total quinolizidine and **(G)** total alkaloids by *Lupinus* cultivar and environment. nd, not detected; <(value), below the limit of quantification. The different letters within each graph mean statistically significant differences (*p* < 0.05).

In the analyzed samples, 32 alkaloids were identified as belonging to three chemical classes (indole, piperidine, and quinolizidine). The list of the identified alkaloids in the studied *Lupinus* cultivars is presented in **Table S2**. All the classes were affected (*p* < 0.05) by the genotype × environment interaction (**Table 3**), which accounted for 25% to 42% of the variability of the results. It was also observed that the genotype was the main source of variation (48% to 74%) of the alkaloid content in *Lupinus* seeds (**Table 3**). The results of the total content of alkaloids and by chemical class are presented in **Figure 2**. Considering the different classes of alkaloids, the most relevant ones were the quinolizidine-based compounds, followed by the indoles and piperidines. The indole alkaloids were only present in *L. luteus* cultivars Acos and Cardiga, with the highest contents being found in the Cardiga cultivar in MI year 2 and VR year 2 (624 mg kg^−1^ DM and 581 mg kg^−1^ DM, respectively) and the lowest in the same cultivar in MI year 1 and VR year 1 (21.4 mg kg^−1^ DM and 14.5 mg kg^−1^ DM, respectively; **Figure 2A**). The piperidine alkaloids were quantified in *L. albus* cultivars and *L. luteus* cultivars Acos, Alburquerque, and Cardiga (**Figure 2B**). In the remaining cultivars piperidine alkaloids were either not detected or not quantified. The highest piperidine alkaloids value was found for *L. luteus* cv. Cardiga in MI year 1 (99.9 mg kg^−1^ DM), followed by the same cultivar in VR year 1 (55.4 mg kg^−1^ DM). The bicyclic quinolizidines were only quantified in *L. luteus* cultivars, with the highest being in *L. luteus* cv. Cardiga in MI year 2 and VR year 2 (1,531 mg kg^−1^ DM and 1,437 mg kg^−1^ DM, respectively; **Figure 2C**), followed by *L. luteus* cv. Acos in the same environments (909 mg kg^−1^ DM and 843 mg kg^−1^ DM; **Figure 2C**). *L. albus* and *L. angustifolius* were the species containing detectable levels of tricyclic quinolizidine alkaloids (**Figure 2D**), with the highest values being found for Estoril and Tango cultivars in MI year 1 and VR year 1 (90.7 mg kg^−1^ DM –265 mg kg^−1^ DM) and the lowest in *L. albus* cv. Estoril in MI year 2 and VR year 2 (54.9 mg kg^−1^ DM and 62.1 mg kg^−1^ DM, respectively). Tetracyclic quinolizidine alkaloid occurrence was verified in all the studied lupin cultivars (**Figure 2E**). *L. luteus* cv. Cardiga showed the highest concentration in MI year 1 (331 mg kg^−1^ DM), followed by the same cultivar in VR year 1 (265 mg kg^−1^ DM), and the lowest levels were obtained for *L. luteus* cv. Mister (3.23 mg kg^−1^ DM –14.8 mg kg^−1^ DM; **Figure 2E**). The total quinolizidine alkaloid content was the highest for *L. luteus* cv. Cardiga (473 mg kg^−1^ DM 1,746 mg kg^−1^ DM; **Figure 2F**), and *L. luteus* cv. Acos in MI year 2 and VR year 2 (1,097 mg kg^−1^ DM and 1,022 mg kg^−1^ DM, respectively). As the major class of alkaloids in *Lupinus* seeds are quinolizidines, it was verified that the same pattern was recorded for the total alkaloid content (**Figure 2G**).

**Table 3:** Analysis of variance of the concentrations (mg kg^−1^ DM) of indole, piperidine, quinolizidine (bicyclic, tricyclic, tetracyclic, and total), and total alkaloids for *Lupinus* cultivars sown in four environments (with a combination of sowing locations and years)*.

Source of variation	df	*Total alkaloids*				*Indole*				*Piperidine*			
		SS		*p*-value	%TRT	SS		*p*-value	%TRT	SS		*p*-value	%TRT
Genotype	6	26,504,796		< 0.001	65	1,281,631		< 0.001	48	21,473		< 0.001	48
Environment	3	2,388,259		< 0.001	6	231,129		< 0.001	9	4,670		0.002	10
Rep(environment)	12	557,096				10,540			0	2,077			
Genotype *×* environment	18	12,130,509		< 0.001	30	1,140,504		< 0.001	43	18,687		< 0.001	42
Residuals	68	4,258,864				25,488				11,592			
Source of variation	df	Quinolizidines											
		*Bicyclic*			*Tricyclic*			*Tetracyclic*			*Total*		
		SS	*p*-value	%TRT	SS	*p*-value	%TRT	SS	*p*-value	%TRT	SS	*p*-value	%TRT
Genotype	6	12,461,805	< 0.001	62	292,535	< 0.001	49	606,520	< 0.001	74	15,533,870	< 0.001	64
Environment	3	2,281,305	< 0.001	11	109,195	< 0.001	18	6,749	0.570	1	1,268,024	0.001	5
Rep(environment)	12	439,274			21,682			38,563			506,176		
Genotype *×* environment	18	5,431,475	< 0.001	27	189,369	0.010	32	202,688	< 0.001	25	7,438,762	< 0.001	31
Residuals	68	2,844,575			322,369			227,706			3,950,639		

*df, degree of freedom; SS, sum of squares; TRT, total sum of squares relative to main effects (TRT = SS_Environment_ + SS_Genotype_ + SS_Genotype × environment_).

### 3.3 Content of individual alkaloids

A total of 13 individual alkaloids, identified in the analyzed *Lupinus* samples (**Table S3**), were quantified. All the individual alkaloids were affected (*p <*0.05) by the interaction genotype × environment (**Table 4**).

**Table 4:** Analysis of variance of the concentrations (mg kg^−1^ DM) of individual alkaloids for *Lupinus* cultivars sown in four environments (with a combination of sowing locations and years)*.

Source of variation	df	Indole						Piperidine								
		Gramine			Gramine derivative			Smipine			*N*-methylammodendrine			Ammodendrine		
		SS	*p*-value	%TRT	SS	*p*-value	%TRT	SS	*p*-value	%TRT	SS	*p*-value	%TRT	SS	*p*-value	%TRT
Genotype	6	1,190,557	< 0.001	48	1,687.71	< 0.001	43	444.32	< 0.001	69	4,048.6	< 0.001	41	6,913.1	< 0.001	52
Environment	3	214,601	< 0.001	9	333.14	< 0.001	9	50.79	0.001	8	916.3	0.008	9	1,143.6	0.015	9
Rep(environment)	12	9,331			63.49			20.13			577.1			862.7		
Genotype × environment	18	1,053,810	< 0.001	43	1,869.62	< 0.001	48	145.48	< 0.001	23	4,884.2	< 0.001	50	5,328.1	< 0.001	40
Residuals	68	22,321			289.54			182.06			3,213.9			4,940		
Source of variation	df	Bicyclic			Tricyclic						Tetracyclic					
		Lupinine			Angustifoline			11,12-seco-12,13-didehydromultiflorine		Multiflorine				Sparteine		
		SS	*p*-value	%TRT	SS	*p*-value	%TRT	SS	*p*-value	%TRT	SS	*p*-value	%TRT	SS	*p*-value	%TRT
Genotype	6	12,461,805	< 0.001	62	131,775	< 0.001	40	140,404	< 0.001	71	405.24	< 0.001	60	834,822	< 0.001	86
Environment	3	2,281,305	< 0.001	11	56,550	< 0.001	17	8,937	0.102	4	48.17	0.14	7	1,862	0.665	0
Rep(environment)	12	439,274			17,125			13,867			87.3			13,842		
Genotype × environment	18	5,431,475	< 0.001	27	142,423	< 0.001	43	49,282	0.006	25	221.14	0.136	33	128,987	< 0.001	13
Residuals	68	2,844,575			160,437			78,753			574.27			80,788		
Source of variation	df	Tetracyclic														
		11,12-dehydrosparteine			Lupanine			α-iso-lupanine			13-α-hydroxylupanine			13-α-angelolyoxylupanine		
		SS	*p*-value	%TRT	SS	*p*-value	%TRT	SS	p-value	%TRT	SS	*p*-value	%TRT	SS	*p*-value	%TRT
Genotype	6	105.07	< 0.001	49	136,832	< 0.001	80	482.19	< 0.001	79	56,779	< 0.001	70	386.47	< 0.001	49
Environment	3	18.86	< 0.001	9	8,427	0.006	5	39.24	0.002	6	2,299	0.228	3	122.47	< 0.001	15
Rep(environment)	12	1.13			4,973			16.7			5,537			36.26		
Genotype × environment	18	91.48	< 0.001	42	26,763	0.037	16	89.61	0.36	15	22,587	< 0.001	28	284.02	< 0.001	36
Residuals	68	7.55			54,746			304.38			24,897			199.33		

*df, degree of freedom; SS, sum of squares; TRT, total sum of squares relative to main effects (TRT = SS_Environment_ + SS_Genotype_ + SS_Genotype × environment_).

**Figure 3:**
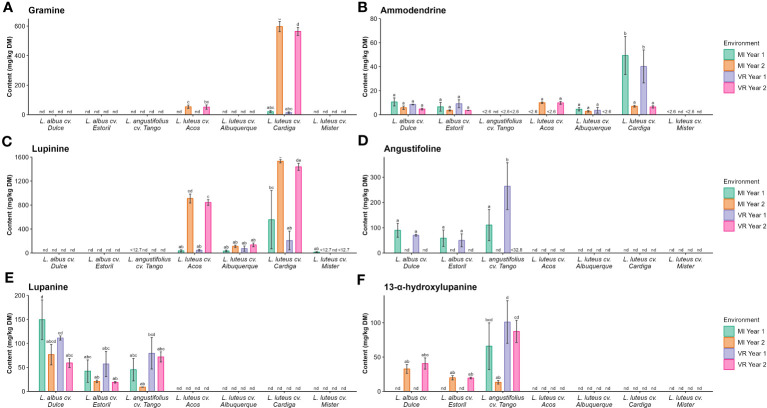
Concentrations (mg kg^−1^ DM) of **(A)** gramine, **(B)** ammodendrine, **(C)** lupinine, **(D)** angustifoline, **(E)** lupanine, and **(F)** 13-α-hydroxylupanine by *Lupinus* cultivar and environment. nd, not detected; <(value), below the limit of quantification. The different letters within each graph mean statistically significant differences (*p* < 0.05).

The complete results for the quantification of these alkaloids are presented in **Table S3**. Gramine was measured in *L. luteus* cv. Acos and *L. luteus* cv. Cardiga, the highest contents were found in the former for MI year 2 (596 mg kg^−1^ DM) and VR year 2 (564 mg kg^−1^ DM), as verified for the gramine derivative (27.7 mg kg^−1^ DM and 16.9 mg kg^−1^ DM, respectively, **Figure 3A**). Smipine characterized the *L. albus* cultivars, with the highest content recorded in *L. albus* cv. Dulce MI year 1 (11.1 mg kg^−1^ DM; **Table S3**). *N*-methylammodendrine was present only in *L. luteus* cv. Cardiga, with the highest levels recorded in MI year 1 (49.4 mg kg^−1^ DM). Ammodendrine (**Figure 3B**) was present in *L. albus* cultivars, and *L. luteus* cultivars Acos, Alburquerque, and Cardiga, with the Cardiga showing the highest content for MI year 1 (49.3 mg kg^−1^ DM) and VR year 1 (40.2 mg kg^−1^ DM). Lupinine was only detected in *L. luteus* cultivars (**Figure 3C**), while angustifoline (**Figure 3D**), lupanine (**Figure 3E**), and 13-α-hydroxylupanine (**Figure 3F**) were only present in the *L. albus* and *L. angustifolius* cultivars.

### 3.4 Effect of the environment on the productivity and alkaloid content

The stability of the cultivars across years and locations (i.e., environments) was evaluated in terms of productivity (i.e., seed and protein production) and alkaloid content. For the latter, only total alkaloid content was evaluated. The results of the ANOVA (**Tables 2**, **3**) showed that the interaction genotype × environment was significant (*p* < 0.05) for seed and protein production, and for total alkaloid content. In terms of productivity indexes, the main source of variation was the environment, accounting for 73% and 74% of the total sum of squares relative to the main effects (%TRT) for seed and protein production, respectively. On the other hand, the main source of variation in alkaloid content was caused by the genotype, with %TRT values of 65%, followed by the interaction genotype × environment (30%).

The stability of the cultivars in terms of seed and protein production revealed that the most stable cultivar across environments was *L. angustifolius* cv. Tango (**Table 5**) by the three indexes for which the lower the value, the higher the stability. This cultivar was followed by *L. luteus* cv. Acos and *L. luteus* cv. Alburquerque. Although the highest productivity values were obtained for *L. albus* cv. Estoril (seed: 2.42 t DM ha^−1^; and protein: 0.909 t DM ha^−1^), it was considered the least stable genotype.

**Table 5:** Stability parameters of the seven *Lupinus* cultivars across environments*.

	Seeds				Protein				Total alkaloids			
	Mean	CV	W	Shukla	Mean	CV	W	Shukla	Mean	CV	W	Shukla
*L. albus* cv. Dulce	1.77	93.6	5	5	0.693	95.7	5	5	207	34.0	3	3
*L. albus* cv. Estoril	2.42	97	7	7	0.909	96.1	7	7	198	54.6	4	4
*L. angustifolius* cv. Tango	1.73	70.1	1	1	0.564	72.9	1	1	229	78.4	5	5
*L. luteus* cv. Acos	1.46	91.9	2	2	0.575	96.1	2	2	630	103.0	6	6
*L. luteus* cv. Alburquerque	1.46	76.9	3	3	0.593	80.5	3	3	130	25.0	1	1
*L. luteus* cv. Cardiga	1.20	78.5	4	4	0.466	76.6	4	4	1,540	58.7	7	7
*L. luteus* cv. Mister	1.04	46.4	6	6	0.394	45.2	6	6	19.5	41.5	2	2

*CV, coefficient of variation; W, rank of the ecovalence stability index (Wricke, 1962; Wricke, 1964); Shukla, rank of the Shukla’s variance (Shukla, 1972).

The content of total alkaloids was found to be more stable across environments in *L. luteus* cv. Alburquerque, and *L. luteus* cv. Mister, corresponding also to the cultivars with the lowest total alkaloid content (130 mg kg^−1^ DM and 19.5 mg kg^−1^ DM, respectively; **Table 5**). On the other hand, *L. luteus* cv. Cardiga with the highest mean value for alkaloid content (1,540 mg kg^−1^ DM) was considered the least stable genotype.

**Figure 4:**
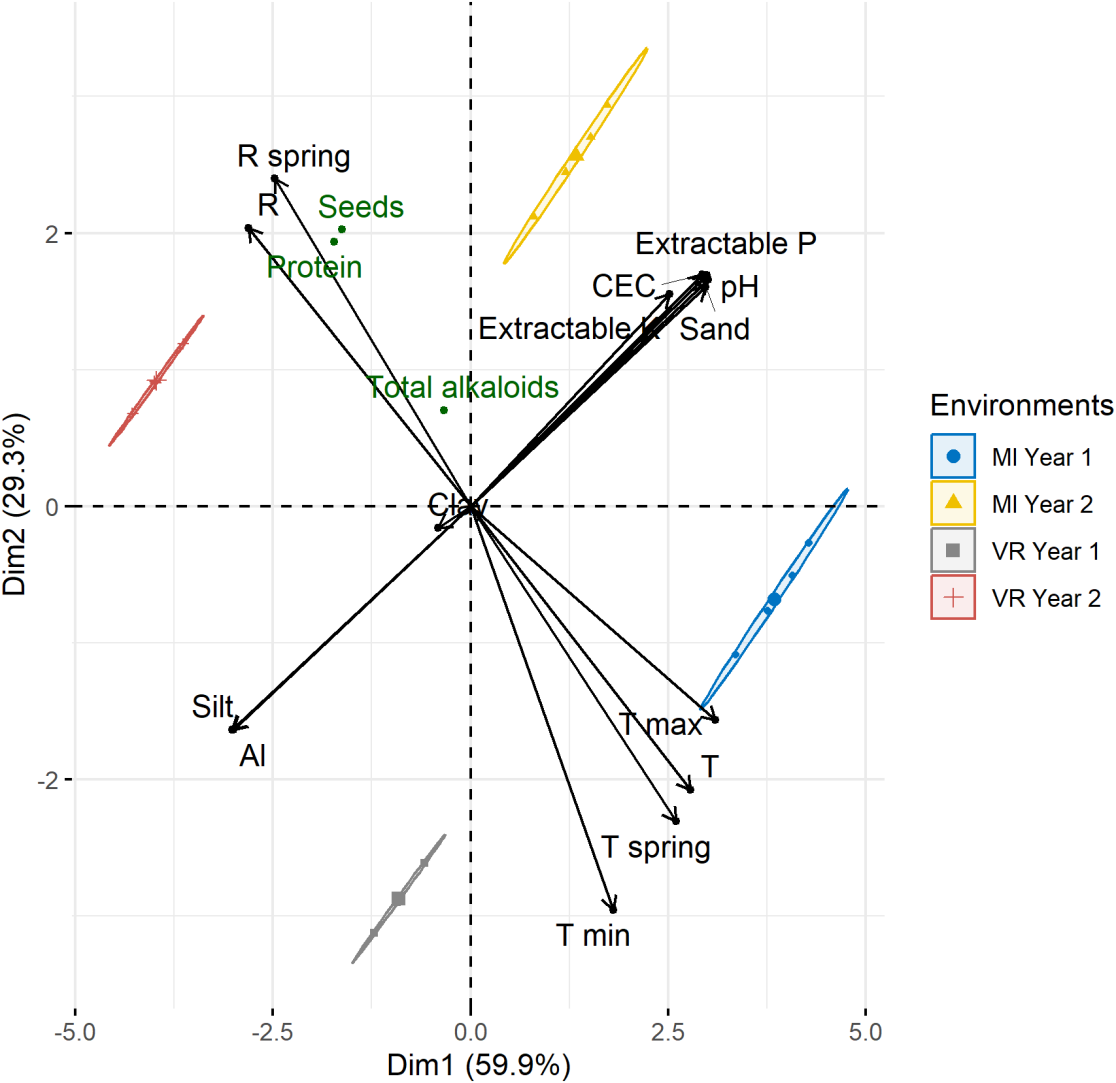
Principal component analysis (PCA) biplot showing the environments and the environmental variables.

The correlation patterns between the edaphoclimatic variables and the differences between environments was studied by the PCA biplot, as presented in **Figure 4**. The first two components (PC) accounted for 89.2% of the total variability of the biplot (**Figure 4**), thus highlighting the differences between the environments. The variables were clustered into two main groups: (1) the climatic variables (temperature and rainfall) that distinguish the sowing years; and (2) the characteristics of soil that discriminated the locations. In each of these groups all the variables were highly correlated between them (*r* > 0.75), except clay. It was also possible to verify that seed and protein production was highly affected by the higher rainfall values in year 2.

**Figure 5:**
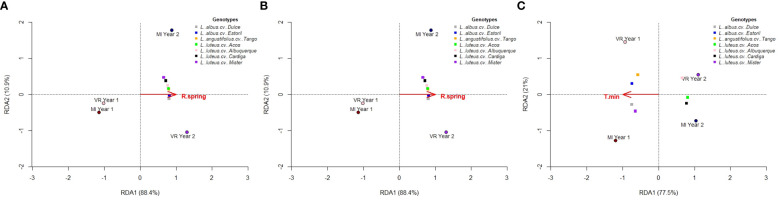
Reduced rank factorial regression biplots for **(A)** seed production, **(B)** protein production, and **(C)** total alkaloid content.

The environmental variable with a significant impact on productivity, as selected for by the RDA procedure, was the total spring rainfall (R spring) for seed (**Figure 5A**) and protein (**Figure 5B**) production (*p* = 0.042, model *R* ^2^ = 0.884 for seed and model *R* ^2^ = 0.926 for protein). The biplots presented in **Figures 5A, B** shows the relationship between the environmental variables, the environment scores, and the genotype scores, with the first two axes accounting for 99.3% of the total genotype by environment variation. The total spring rainfall was positively correlated with year 2 at both locations, and with the increased seed and protein production in the same environments. For total alkaloid content, no environmental variable was considered to significantly (*p* < 0.05) affect the alkaloid production. In addition, the mean minimum temperature (T min) was shown to have a low impact on the alkaloids’ concentration (*p* = 0.083, model *R* ^2^ = 0.775), and the results are depicted in **Figure 5C**. Higher T min was associated with the environments MI year 1 and VR year 1, and this had an impact on the highest content of alkaloids for *L. albus* cv. Dulce, *L. albus* cv. Estoril, *L. angustifolius* cv. Tango, and *L. luteus* cv. Mister. For total quinolizidine alkaloids, the same results were obtained (data not shown).

## 4 Discussion

### 4.1 Productivity and seed chemical composition

The *Lupinus* productivity was affected by the interaction genotype × environment, and a marked impact of the environment was verified by the ANOVA for the genotype-by-environment interaction model. The differences on the productivity were essentially observed in *L. albus* cultivars (Dulce and Estoril), *L. angustifolius* cv. Tango, and *L. luteus* cv. Acos, for which the productivities were higher in year 2 at both locations. In fact, *L. albus* cultivars were ranked as genotypes with low stability across environments, although average higher mean values of seed and protein production were obtained, as reported by other authors (Jul et al., 2003; Gresta et al., 2010). On the other hand, *L. angustifolius* cv. Tango and *L. luteus* cv. Acos were the two most stable cultivars, indicating a better adaptability to the edaphoclimatic conditions of the trial, when compared to the remaining cultivars. Although French et al. (2001) reported higher grain yields (around 51%) for *L. angustifolius* than *L. luteus*, in the present work, the production of *L. angustifolius* cv. Tango was similar to that of *L. luteus* cultivars. When studying two *L. angustifolius* cultivars Borweta and Bordako, Fraser et al. (2005) observed very different grain production values (3 t DM ha^−1^ for Borweta and 1.5 t DM ha^−1^ for Bordako), showing the high variability in productivity within the species. The grain productions of *L. luteus* cultivars were within the broad range of values reported by other authors (Chiofalo et al., 2012; Grażyna et al., 2017).

The differences in production yields are described to have been influenced by the climatic conditions (Maknickiene, 2001; Jul et al., 2003), and the soil characteristics, such as the pH (Gresta et al., 2010) and the phosphorous and aluminum concentrations (Quiñones et al., 2022). In the present study, it was observed that the higher total rainfall during spring (between March and May) observed in year 2 at both locations was responsible for the increased productivity. In Mediterranean countries, due to the low temperatures observed during the early stages of the vegetative phase (between November and February), the main growth stage of the plants occurs between March and May. The rainfall distribution during the vegetative cycle of *Lupinus* is recognized as having an important role on the *Lupinus* seed yield (Perdigão et al., 2021) as verified in the present work.

The seeds’ chemical composition was essentially affected by *Lupinus* cultivars, except for ash, which was significantly affected by the genotype × environment interaction, as also reported by Calabrò et al. (2015). Higher crude protein levels were observed for *L. luteus* cultivars (42.7 g 100 g^−1^ DM –44.4 g 100 g^−1^ DM) and *L. albus* cv. Dulce (42.6 g 100 g^−1^ DM), followed by *L. albus* cv. Estoril (37.8 g 100 g^−1^ DM), and *L. angustifolius* cv. Tango (32.2 g 100 g^−1^ DM). These values were higher than those reported by Gresta et al. (2010) for *L. luteus*, but within the values found by other authors (Yu et al., 2002; Beyer et al., 2015). The ether extract values were the highest and neutral detergent fiber the lowest for *L. albus* cultivars Dulce and Estoril, and the values were within the range reported for this species (Calabrò et al., 2015).

### 4.2 Effects of cultivar and edaphoclimatic conditions on the seed alkaloid content

A high variability of the total alkaloid content was observed with a significant effect of the genotype × environment, similar results were also reported by Calabrò et al. (2015) for *L. albus* cultivars and by Beyer et al. (2015) for *L. angustifolius*. The increased accumulation of alkaloids in lupins seeds has been associated with edaphoclimatic variables. Jansen et al. (2009) observed a 3.7-fold increase in the total alkaloids in *L. angustifolius* for an increase of about 3°C in the daily mean temperature, that is, a small increase in ambient temperature can cause a drastic increase in the production of alkaloids. Moreover, drought stress can be also responsible for the production of higher levels of alkaloids in seeds (Christiansen et al., 1997). The soil characteristics, such as pH, the elemental composition, and the type and amount of fertilizer used, can also impact alkaloid production in *Lupinus* plants.

In our study, total alkaloid content increased in year 2 for Cardiga and Acos *L. luteus* cultivars, with no differences between environments for the other cultivars, suggesting a different reaction of these cultivars to environmental conditions. *L. luteus* cv. Cardiga and Acos total alkaloid content has shown higher variability between environments than the other cultivars, which was confirmed by the lowest stability indexes for these genotypes across environments, thus suggesting that alkaloid accumulation in the seeds in these cultivars is dependent on the edaphoclimatic conditions. No explanation for the differences between these genotypes and the remaining in the study was found elsewhere, but it can be supposed that these cultivars show a distinct response to abiotic stress conditions caused by different profiles of temperature and rainfall during the different stages of the plant development. The environmental variables analysis by RDA showed that the mean minimum temperature in year 1 for both sowing locations had some impact on the reduced production of alkaloids in these environments. Considering the safety limit of 200 mg kg^−1^, which is accepted by many health authorities for the direct use of lupins in feed and food products (Pilegaard and Gry, 2009), only *L. luteus* cv. Alburquerque and Mister contained less than this value in both sowing years.

Total alkaloid content reflects the content of quinolizidine alkaloids, often known as “lupin alkaloids”, the most relevant ones in the *Lupinus* genus (Frick et al., 2017), thus deserving more attention in the context of antinutritional compounds in lupins, with most of the scientific publications and regulatory documents describing only this category of alkaloids. This alkaloid class occurs mostly in the Fabaceae family and is responsible for the protection of plants against insect pests (Philippi et al., 2015). The biosynthesis of quinolizidine alkaloids occurs via decarboxylation of L-lysine, forming the major structural compounds lupinine (bicyclic), sparteine, lupanine, and multiflorine (tetracyclic). These alkaloids can be further modified by dehydrogenation, oxygenation, hydroxylation, glycosylation, or esterification forming a wide variety of related quinolizidines (Frick et al., 2017), as those identified in the present work.

The environmental impact on the seed alkaloid content is recognized to be high, affecting the compounds’ biosynthesis and transport from the plant tissues to the seed (Frick et al., 2017). The quinolizidine biosynthesis is regulated by light and water conditions, with production being increased during the day and in conditions of low water. Although the effects of drought in *Lupinus* seeds are unclear and unpredictable (Christiansen et al., 1997), the increase in the temperature has been reported to have a significant impact on quinolizidine alkaloid production in seeds of narrow-leafed cultivars (Cowling and Tarr, 2004; Jansen et al., 2009; Frick et al., 2017; Frick et al., 2018), although no effect was observed in the present work. The soil characteristics also play a role on quinolizidine alkaloid content in lupin seeds, as lower soil pH and potassium deficiency increases the levels of quinolizidine alkaloids in seeds (Jansen et al., 2012), whereas phosphorous deficiency reduces them (Gremigni et al., 2003).

Indole and piperidine alkaloids are also present in *Lupinus* seeds along with quinolizidine alkaloids, although often disregarded. A detailed characterization of the structural diversity of alkaloids produced by lupins is pivotal to effectively assessing the full potential of *Lupinus* seed cultivars, as toxic effects may be expected especially relating to the indole/piperidine/quinolizidine nucleus. This information is important both for selecting the cultivars better adapted to biotic stresses during plant growth and the ones best suited for food and feed purposes. The high levels of quinolizidine alkaloids in *Lupinus* seeds gives them a bitter taste and this is a health concern due to their high toxicity (Schrenk et al., 2019). In this sense, most of the studied lupin cultivars should be processed for use in food purposes through specific debittering processes (between 89% and 97% of quinolizidine alkaloids present in seeds are removed by water treatment and boiling), and its inclusion level must be low when used in animal feeds (Schrenk et al., 2019). The only exceptions were the yellow lupin cultivars Alburquerque and Mister. The *Lupinus* species can be distinguished based on their individual alkaloid profile, agreeing with previously described alkaloid profiles for each species (Wink et al., 1995; Boschin et al., 2008; Calabrò et al., 2015; Magalhães et al., 2017; Osorio et al., 2018; Święcicki et al., 2019). Tricyclic quinolizidines were characteristic of *L. albus* and *L. angustifolius*, while angustifoline was the main alkaloid present in *L. angustifolius* cv. Tango, with it being detected at more than twice the content of that recorded in *L. albus*, with 11,12-seco-12,13-didehydromultiflorine only being detected in the *L. albus* species. Although 11,12-seco-12,13-didehydromultiflorine is structurally a tricyclic compound, its occurrence in plants is related to its precursor multiflorine, a tetracyclic quinolizidine, also only quantified in the *L. albus* samples. Wink et al. (1995) reported that these tri- and tetracyclic quinolizidine alkaloids have a restricted distribution over the *Lupinus* species, with *L. albus* being one of the species in which these alkaloids are produced. The occurrence of lupanine and related alkaloids (α-iso-lupanine, 13α-hydroxylupanine, and 13-α-angelolyoxylupanine) were essentially limited to *L. albus* and *L. angustifolius*, the profile varied between the species. Lupanine is considered to be one of the most toxic alkaloids to humans and animals (Schrenk et al., 2019), and has a large impact on aphid survival (Ridsdill-Smith et al., 2001).

The presence and formation of smipine, a piperidine alkaloid, in lupins is poorly reported. Wink et al. (1995) reported the identification of this alkaloid in the *Lupinus* genus; however, no results of its quantification were shown. Magalhães et al. (2017) determined low levels of this alkaloid in some *L. albus* cultivars (10 mg kg^−1^ DM–30 mg kg^−1^ DM), but not in *L. angustifolius* or *L. luteus*. In the present work, smipine was quantified in *L. albus* cv. Dulce and Estoril. Interestingly, the information about smipine occurrence in plants is very scarce; it is described to be present in *Lupinus formosus* (Fitch et al., 1974), the desert plant *Haloxylon salicornicum* (El-Shazly et al., 2005), and as one of the major alkaloids of the genus *Dichilus* (Van Wyk et al., 1988).

Lupinine, the most abundant alkaloid in *L. luteus* (Wink et al., 1995; Święcicki et al., 2019), was quantified in the range 14.1 mg kg^−1^ DM to 1,531 mg kg^−1^ DM, with the Acos and Cardiga cultivars being those with the highest contents. The strong insecticidal activity of lupinine has been reported (Campbell et al., 1933). Sparteine, which is two or three times more toxic to animals than lupanine (Schrenk et al., 2019), was produced at levels between 9% and 80% of the total alkaloid content in *L. luteus* cultivars (3.23 mg kg^−1^ DM–331 mg kg^−1^ DM). Sparteine toxicity to humans is largely studied due to its use in the past as an antiarrhythmic and oxytocic drug (Schrenk et al., 2019).

Gramine, rare in the *Lupinus* species, was quantified in *L. luteus* bitter cultivars (with high total alkaloid levels) Acos (51.6 mg kg^−1^ DM–53.8 mg kg^−1^ DM) and Cardiga (14.5 mg kg^−1^ DM–596 mg kg^−1^ DM). Besides, a high increase on the seeds' gramine content was observed in year 2 when compared to year 1 suggesting a high influence of the edaphoclimatic conditions on the production of this alkaloid. Similarly, gramine has been reported to occur in *L. luteus*, and not in *L. albus* or *L. angustifolius* (Magalhães et al., 2017; Święcicki et al., 2019). Indeed, only some *L. luteus* cultivars are known to produce gramine (Osorio et al., 2018), and earlier studies have shown that the introduction and cultivation of *L. luteus* throughout Central Europe resulted in a considerable decrease of the gramine content, as opposed to the wild forms of this species (Święcicki and Jach, 1980). From the nutritional perspective, the high levels of this alkaloid in Acos and Cardiga cultivars is not desirable, yet it can be important in plant defense mechanisms, as gramine was found to be one of the most toxic compounds to aphids, after lupanine (Ridsdill-Smith et al., 2001).

Ammodendrine was present in the three lupin species, ranging in concentration from 0.3% to 7.4% of the total alkaloid content. The high levels of ammodendrine found may preclude the direct use of the white, narrow-leafed, and yellow lupins cultivars studied for food and feed purposes, as this alkaloid is suspected to be a teratogen for both humans and livestock (Green et al., 2012).

The great discrepancy in the alkaloid content among cultivars, and also among years, either total and by chemical class, may indicate cross-pollination with spontaneous lupins increasing alkaloid levels in some cultivars (Boschin and Resta, 2013). In fact, the ancestors of *L. luteus* (and *L. angustifolius*) originated in the Iberian Peninsula and, although the origin of *L. albus* is not known, semi-wild cultivars of these species have been cultivated throughout the entire Mediterranean region over a long period of time (Gustafsson and Gadd, 1965). The levels of alkaloids dropped considerably during lupins’ domestication, but in the wild populations alkaloids can reach very high levels of concentration (Gustafsson and Gadd, 1965; Otterbach et al., 2019). For instance, up to 12% of the seed DM of quinolizidine alkaloids were found in wild populations of *L. albus* and 1.5% of total alkaloids (including gramine) were described in a semi-domesticated Spanish accession of *L. luteus* (Osorio et al., 2018). Also, many papers do not report piperidine alkaloids. Although most *Lupinus* species produce trace levels of these alkaloids (Wink et al., 1995), the present study suggests that this class may contribute for the observed differences among the cultivars.

## 5 Conclusions

The productivity and alkaloid content of *Lupinus* seeds showed to be affected by the environment, with a distinct impact in different cultivars. *Lupinus angustifolius* cv. Tango, *L. luteus* cv. Acos, and *L. luteu*s cv. Alburquerque were the most stable cultivars across the environments in terms of seed and protein production, in opposition to *L. albus* cv. Estoril, the most productive cultivar but with the least stability. The productivity was mainly affected by the total rainfall during the spring months, which correspond to the vegetative phase of the plant; higher rainfall in the second year of the trial at both locations resulted in higher seed and protein production. For the total alkaloid content, *L. luteus* cv. Alburquerque and *L. luteus* cv. Mister were the genotypes with the highest stability and the lowest alkaloid content. *Lupinus luteus* cv. Cardiga seeds were those with the highest alkaloids levels and the lowest stability across environments. The increased accumulation of alkaloids in *L. albus* cv. Dulce, *L. albus* cv. Estoril, *L. angustifolius* cv. Tango, and *L. luteus* cv. Mister seeds in the first year of the study could be partially attributed to the higher minimum temperatures observed in those environments. Still, this finding should be carefully studied as it is postulated that the high levels of genetic variability of these cultivars can lead to different metabolic responses in the plants and, consequently, different responses to abiotic stress through alkaloid biosynthesis.

The high total alkaloid content of most of the cultivars studied limits its consumption as raw food, as a safety limit of 200 mg kg^−1^ in seeds for human consumption is recommended by several health authorities. In this context, only *L. luteus* cv. Alburquerque and Mister presented alkaloid values below this limit, implying that the other cultivars should not be consumed before alkaloid removal. Due to the high alkaloid content of some of the studied cultivars conferring resistance to pests, they can be particularly important for recovering poor or exhausted soils due to their ability to grow in highly infertile, neutral to acidic soils, thus enriching them in nitrogen and mobilizing phosphorus that can be used by non-legumes in crop rotations.

## Data availability statement

The raw data supporting the conclusions of this article will be made available by the authors, without undue reservation.

## Author contributions

IV, CS, MA, and MM conducted the analytical work. MA, VP, CM, and SG-S conducted the field study and sample collection. LF and CG were involved in the proximate composition analysis. AF and HT conceived and designed the experimental study. IV and CS analyzed the data. IV, MM, AC, and AF performed the statistical analysis. IV wrote the first draft of the manuscript. HT conceived and designed the field study. All authors contributed to the article and approved the submitted version.
