# Supplementary material for: Insights from the yield, protein production, and detailed alkaloid composition of white (Lupinus albus), narrow-leafed (Lupinus angustifolius), and yellow (Lupinus luteus) lupin cultivars in the Mediterranean region
Source: Front Plant Sci. 2023 Dec 15;14:1231777. doi: 10.3389/fpls.2023.1231777 (PMC10755673; doi:10.3389/fpls.2023.1231777)
Supplement: Supplementary file 1 [file DataSheet_1.pdf]

Supplementary Information

Insights from the yield, protein production, and detailed alkaloid composition of white (*Lupinus albus*), narrow-leafed (*Lupinus angustifolius*), and yellow (*Lupinus luteus*) lupin cultivars in the Mediterranean region

Inês M. Valente*, Carla Sousa, Mariana Almeida, Carla Miranda, Victor Pinheiro, Sofia Garcia-Santos, Luís M. M. Ferreira, Cristina M. Guedes, Margarida R. G. Maia, Ana R. J. Cabrita, António J. M. Fonseca, Henrique Trindade

*** Correspondence:** Inês M. Valente: ines.valente@fc.up.pt

1. Figures

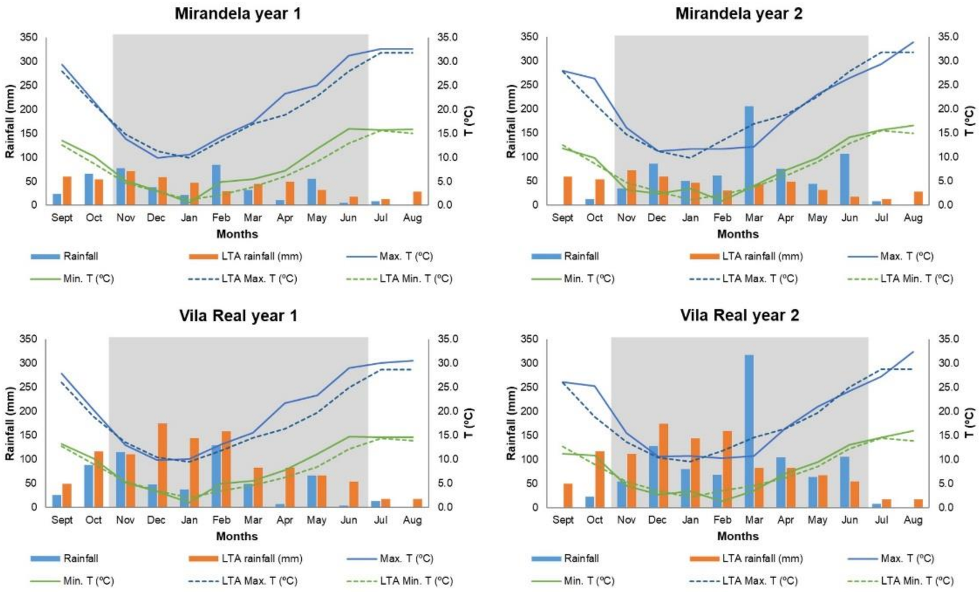

Figure S1. Monthly average minimum and maximum temperatures (˚C) and rainfall (mm) observed in Mirandela and Vila Real and between September and August of both years of study. Long-term averages (LTA, between 1971 and 2000) are also presented. The grey highlighted regions show the sowing period.

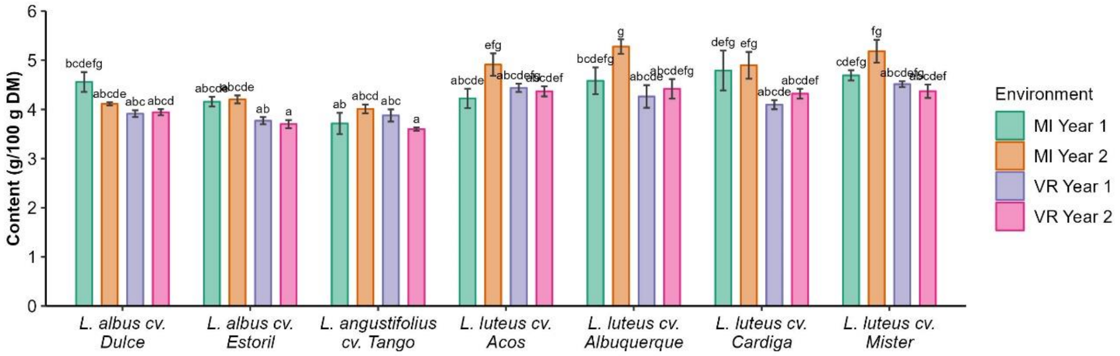

Figure S2. Ash content (g 100 g^{-1} DM) of *Lupinus* seeds by cultivar and environment. Different letters show statistically significant differences ($p < 0.05$).

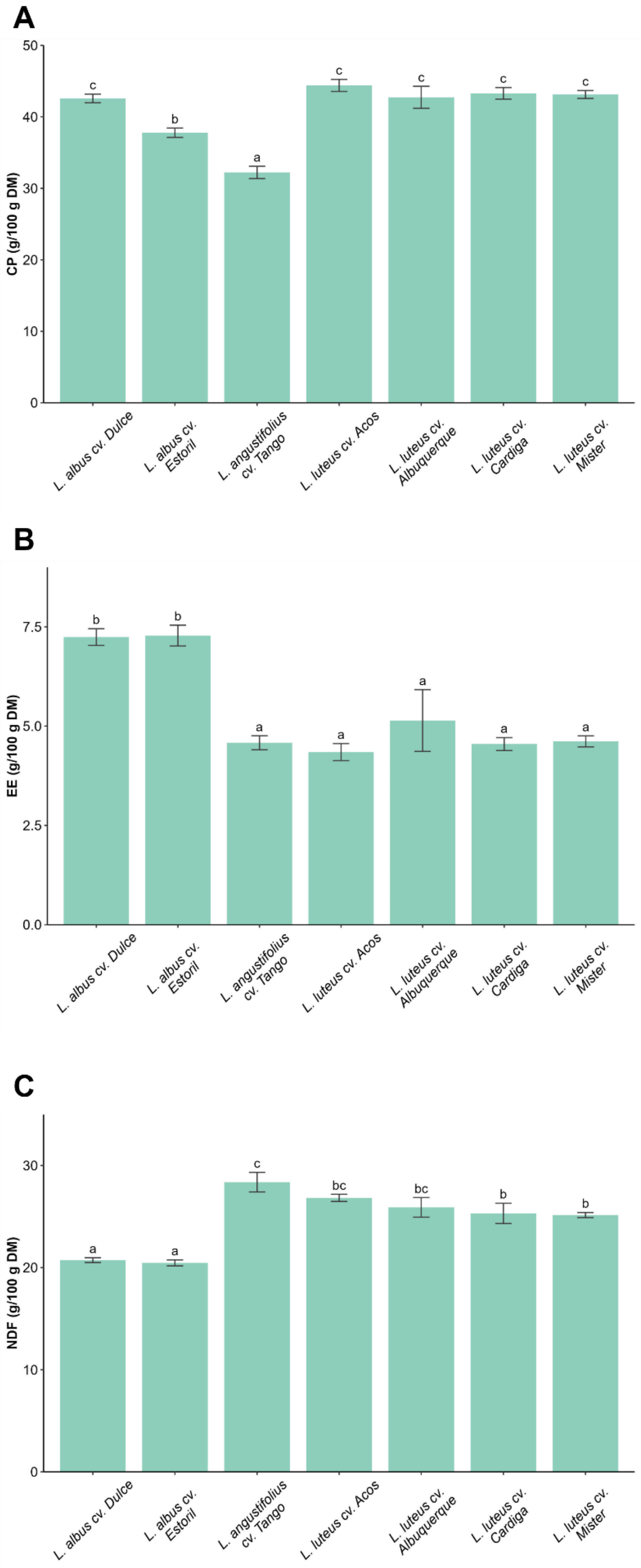

Figure S3. Content (g 100 g^{-1} DM) of (A) crude protein, (B) ether extract, and (C) neutral detergent fiber of *Lupinus* seeds by cultivar. Different letters show statistically significant differences ($p < 0.05$).

2. Tables

Table S1. Germination rates (%) and sowing densities (kg ha^{-1} *) of the *Lupinus* cultivars.

Cultivar	2016/2017		2017/2018	
	Germination rate	Sowing density	Germination rate	Sowing density
L. albus cv. Dulce	98	100	100	100
L. albus cv. Estoril	93	100	100	100
L. angustifolius cv. Tango	95	80	100	80
L. luteus cv. Acos	80	70	48	125
L. luteus cv. Alburquerque	34	160	60	100
L. luteus cv. Cardiga	92	60	88	60
L. luteus cv. Mister	98	60	100	60

*ha - hectare

Table S2. List of alkaloids identified in *Lupinus* cultivars grouped by chemical class.

Alkaloid	Detected in
**Indole**	
**Gramine**	*L. luteus* cv. Acos<br>*L. luteus* cv. Cardiga
**Gramine derivative**	*L. luteus* cv. Cardiga
**Piperidine**	
**Smipine**	*L. albus* cv. Dulce<br>*L. albus* cv. Estoril*<br>*L. luteus* cv. Cardiga*
***N*-methylammodendrine**	*L. luteus* cv. Cardiga
**Ammodendrine**	*L. albus* cv. Dulce<br>*L. albus* cv. Estoril<br>*L. angustifolius* cv. Tango*<br>*L. luteus* cv. Acos<br>*L. luteus* cv. Alburquerque<br>*L. luteus* cv. Cardiga<br>*L. luteus* cv. Mister*
***N*-acetylhystrin**	*L. albus* cv. Dulce*<br>*L. albus* cv. Estoril*<br>*L. luteus* cv. Acos*
**Quinolizidine bicyclic**	
**Lupinine**	*L. luteus* cv. Acos<br>*L. luteus* cv. Alburquerque<br>*L. luteus* cv. Cardiga<br>*L. luteus* cv. Mister*
**Acetyl-epilupinine**	*L. luteus* cv. Cardiga *
**Lustitanine**	*L. luteus* cv. Acos*<br>*L. luteus* cv. Cardiga
**Quinolizidine tricyclic**	
**Albine**	*L. albus* cv. Dulce*<br>*L. albus* cv. Estoril *
***iso*-angustifoline**	*L. albus* cv. Dulce*<br>*L. albus* cv. Estoril*<br>*L. angustifolius* cv. Tango*
**Tetrahydrorhombifoline**	*L. albus* cv. Dulce*<br>*L. albus* cv. Estoril*<br>*L. angustifolius* cv. Tango*
**Angustifoline**	*L. albus* cv. Dulce<br>*L. albus* cv. Estoril*<br>*L. angustifolius* cv. Tango
**11,12-seco-12,13-didehydromultiflorine**	*L. albus* cv. Dulce*<br>*L. albus* cv. Estoril
**Quinolizidine tetracyclic**	
**α-iso-sparteine**	*L. luteus* cv. Acos*<br>*L. luteus* cv. Cardiga*
**Sparteine**	*L. albus* cv. Dulce*<br>*L. albus* cv. Estoril*<br>*L. angustifolius* cv. Tango*<br>*L. luteus* cv. Acos<br>*L. luteus* cv. Alburquerque<br>*L. luteus* cv. Cardiga<br>*L. luteus* cv. Mister
**β-iso-sparteine**	*L. luteus* cv. Acos*<br>*L. luteus* cv. Cardiga*
**11,12-Dehydrosparteine**	*L. luteus* cv. Acos
**7-Hydroxy-β -isosparteine**	*L. luteus* cv. Cardiga*
**17-Oxosparteine**	*L. luteus* cv. Acos*
**Dihydromultiflorine**	*L. albus* cv. Dulce*

	L. albus cv. Estoril*
**α -isolupanine**	*L. albus* cv. Dulce<br>*L. albus* cv. Estoril<br>*L. angustifolius* cv. Tango
**Lupanine**	*L. albus* cv. Dulce<br>*L. albus* cv. Estoril<br>*L. angustifolius* cv. Tango
**11,12-dehydrolupanine**	*L. albus* cv. Dulce*
**7-Hydroxylupanine**	*L. albus* cv. Estoril*<br>*L. luteus* cv. Cardiga*
**Multiflorine**	*L. albus* cv. Dulce*<br>*L. albus* cv. Estoril
**17-oxolupanine**	*L. albus* cv. Dulce*<br>*L. albus* cv. Estoril*<br>*L. angustifolius* cv. Tango*
**13α -hydroxylupanine**	*L. albus* cv. Dulce<br>*L. albus* cv. Estoril<br>*L. angustifolius* cv. Tango
**13α -isovaleroyloxylupanine**	*L. albus* cv. Dulce*<br>*L. albus* cv. Estoril*
**13α -Angelolyoxylupanine**	*L. albus* cv. Dulce<br>*L. albus* cv. Estoril
**13α-Tigloyloxylupanine**	*L. albus* cv. Dulce*<br>*L. albus* cv. Estoril*
**13-Tigloyloxymultiflorine**	*L. albus* cv. Dulce*<br>*L. albus* cv. Estoril*

* - denotes below the limit of quantification.

Table S3. Content ± standard error of the mean (mg kg^{-1} DM) of individual alkaloids in *Lupinus* seeds by genotype and environment. Different letters by column show statistically significant differences ($p < 0.05$). nd, not detected; <(value), below the limit of quantification.

Genotype	Environment	Indole		Piperidine			Bicyclic quinolizidines
		**Gramine**	**Gramine derivative**	**Smipine**	***N*-methylammodendrine**	**Ammodendrine**	**Lupinine**
L. albus cv. Dulce	MI Year 1	nd	nd	11.1 ± 3.6^{c}	nd	10.7 ± 3.4^{a}	nd
	MI Year 2	nd	nd	3.99 ± 0.50^{ab}	nd	5.84 ± 1.41^{a}	nd
	VR Year 1	nd	nd	5.79 ± 0.48^{b}	nd	8.53 ± 0.29^{a}	nd
	VR Year 2	nd	nd	<2.6	nd	4.71 ± 0.77^{a}	nd
L. albus cv. Estoril	MI Year 1	nd	nd	2.96 ± 1.65^{ab}	nd	6.62 ± 3.62^{a}	nd
	MI Year 2	nd	nd	<2.6	nd	3.58 ± 0.48^{a}	nd
	VR Year 1	nd	nd	3.33 ± 1.25^{ab}	nd	9.16 ± 3.31^{a}	nd
	VR Year 2	nd	nd	<2.6	nd	3.60 ± 0.14^{a}	nd
L. angustifolius cv. Tango	MI Year 1	<11.0	nd	nd	nd	<2.6	<12.7
	MI Year 2	nd	nd	nd	nd	nd	nd
	VR Year 1	nd	nd	nd	nd	<2.6	nd
	VR Year 2	nd	nd	nd	nd	<2.6	nd
L. luteus cv. Acos	MI Year 1	nd	nd	nd	nd	<2.6	37.1 ± 16.3 ^{ab}
	MI Year 2	53.8 ± 11.2^{c}	nd	nd	nd	9.98 ± 0.68^{a}	909 ± 74 ^{cd}
	VR Year 1	nd	nd	nd	nd	<2.6	46.7 ± 13.7 ^{ab}
	VR Year 2	51.6 ± 14.6^{bc}	nd	nd	nd	9.86 ± 1.22^{a}	843 ± 47 ^{c}
L. luteus cv. Albuquerque	MI Year 1	nd	nd	nd	nd	4.57 ± 1.36^{a}	34.6 ± 13.7 ^{ab}
	MI Year 2	nd	nd	nd	nd	2.80 ± 0.65^{a}	111 ± 19 ^{ab}
	VR Year 1	<11.0	nd	nd	nd	3.71 ± 2.38^{a}	73.7 ± 43.7 ^{ab}
	VR Year 2	<11.0	nd	nd	nd	<2.6	136 ± 30 ^{ab}
L. luteus cv. Cardiga	MI Year 1	21.4 ± 6.6^{abc}	nd	<2.6	49.4 ± 17.3^{b}	49.3 ± 15.9^{b}	557 ± 486 ^{bc}
	MI Year 2	596 ± 35^{d}	27.7 ± 4.5^{b}	nd	2.63 ± 0.28^{a}	7.09 ± 0.76^{a}	1531 ± 28 ^{e}
	VR Year 1	14.4 ± 5.0^{abc}	nd	<2.6	13.8 ± 3.7^{a}	40.2 ± 13.7^{b}	209 ± 156 ^{ab}
	VR Year 2	564 ± 27^{d}	16.9 ± 2.8^{a}	nd	3.57 ± 0.54^{a}	6.49 ± 0.91^{a}	1437 ± 59 ^{de}
L. luteus cv. Mister	MI Year 1	nd	nd	nd	nd	<2.6	14.1 ± 9.3 ^{ab}
	MI Year 2	nd	nd	nd	nd	nd	<12.7
	VR Year 1	nd	nd	nd	nd	<2.6	nd
	VR Year 2	nd	nd	nd	nd	nd	<12.7

Table S3 (cont.)

Genotype	Environment	Tricyclic quinolizidines		Tetracyclic quinolizidines				
		Angustifoline	11,12-seco-12,13-didehydromultiflorine	Sparteine	11,12-dehydrosparteine	Lupanine	13-α-hydroxylupanine	13-α-angelolyoxylupanine
L. albus cv. Dulce	MI Year 1	89.9 ± 27.5[a]	<32.8	nd	nd	149 ± 41 [d]	nd	nd
	MI Year 2	nd	<32.8	nd	nd	76.6 ± 21.4 [abcd]	32.7 ± 6.4 [ab]	11.0 ± 3.4 [b]
	VR Year 1	70.3 ± 3.7[a]	<32.8	<2.1	nd	111 ± 4 [cd]	nd	nd
	VR Year 2	nd	<32.8	nd	nd	59.2 ± 9.5 [abc]	40.6 ± 8.2 [abc]	10.0 ± 2.8 [b]
L. albus cv. Estoril	MI Year 1	58.6 ± 32.5 [a]	126 ± 69 [bc]	<2.1	nd	42.1 ± 23.3 [abc]	nd	nd
	MI Year 2	nd	47.9 ± 8.7 [ab]	nd	nd	20.8 ± 2.6 [ab]	19.9 ± 3.1 [ab]	3.78 ± 0.58 [a]
	VR Year 1	50.0 ± 25.5 [a]	197 ± 72 [c]	2.53 ± 1.44 [a]	nd	57.1 ± 26.3 [abc]	nd	nd
	VR Year 2	nd	56.7 ± 4.7 [ab]	nd	nd	18.9 ± 1.6 [ab]	19.5 ± 1.3 [ab]	4.46 ± 0.50 [a]
L. angustifolius cv. Tango	MI Year 1	111 ± 61 [a]	nd	3.29 ± 1.71 [a]	nd	45.2 ± 23.4 [abc]	65.9 ± 34.0 [bcd]	nd
	MI Year 2	nd	nd	nd	nd	8.99 ± 0.40 [ab]	13.3 ± 2.5 [ab]	nd
	VR Year 1	265 ± 93 [b]	nd	3.36 ± 1.76 [a]	nd	79.3 ± 32.7 [bcd]	101 ± 31 d	nd
	VR Year 2	<32.8	nd	nd	nd	71.8 ± 10.5 [abc]	87.5 ± 16.1 [cd]	nd
L. luteus cv. Acos	MI Year 1	nd	nd	20.7 ± 5.2 [a]	nd	nd	nd	nd
	MI Year 2	nd	nd	183 ± 16 [b]	5.55 ± 0.73 [b]	nd	nd	nd
	VR Year 1	nd	nd	23.2 ± 0.7 [a]	nd	nd	nd	nd
	VR Year 2	nd	nd	173 ± 6 [b]	5.25 ± 0.42 [b]	nd	nd	nd
L. luteus cv. Albuquerque	MI Year 1	nd	nd	48.9 ± 9.9 [a]	nd	nd	nd	nd
	MI Year 2	nd	nd	17.9 ± 3.6 [a]	nd	nd	nd	nd
	VR Year 1	nd	nd	49.1 ± 23.1 [a]	nd	nd	nd	nd
	VR Year 2	nd	nd	25.2 ± 8.2 [a]	nd	nd	nd	nd
L. luteus cv. Cardiga	MI Year 1	nd	nd	331 ± 66 [c]	nd	<2.6	nd	nd
	MI Year 2	nd	nd	214 ± 7 [b]	nd	nd	nd	nd
	VR Year 1	nd	nd	264 ± 43 [bc]	nd	nd	nd	nd
	VR Year 2	nd	nd	199 ± 16 [b]	nd	nd	nd	nd
L. luteus cv. Mister	MI Year 1	nd	nd	13.6 ± 7.4 [a]	nd	nd	nd	nd
	MI Year 2	nd	nd	3.23 ± 0.63 [a]	nd	nd	nd	nd
	VR Year 1	nd	nd	14.8 ± 5.0 [a]	nd	nd	nd	nd
	VR Year 2	nd	nd	<2.1	nd	nd	nd	nd
